# First Record of the Breeding Biology of the Biet's Laughingthrush (*Ianthocincla bieti*) in Southwest China

**DOI:** 10.1002/ece3.72628

**Published:** 2025-12-09

**Authors:** Caiping Chen, Shixiang Fan, Gang Song, Yanhua Qu, Xu Luo

**Affiliations:** ^1^ College of Forestry/Key Laboratory for Conserving Wildlife With Small Populations in Yunnan Southwest Forestry University Kunming China; ^2^ Key Laboratory of Zoological Systematics and Evolution, Institute of Zoology Chinese Academy of Sciences Beijing China; ^3^ College of Biological Science and Food Engineering/Yunnan Academy of Biodiversity Southwest Forestry University Kunming China

**Keywords:** breeding biology, *Ianthocincla bieti*, Laojun mountain, Leiothrichidae, life history characteristics

## Abstract

Breeding biology of the poorly known Biet's Laughingthrush (*Ianthocincla bieti*) was studied in the mixed coniferous and broad‐leaved forests at Laojun mountain in southwest China. We discovered two active nests in 2021 and 2024; both were located on bamboo stands, 3.7 and 4.5 m above the ground respectively. The nest is open, bowl‐ or cup‐shaped, and mainly composed of dry grass stems, bamboo branches and leaves. We made direct observations on parent birds, used infrared camera to monitor the breeding events in the nest, and also made video recordings of the incubation and brooding behavior. Both parent birds incubated the eggs and provisioned the nestlings. The incubation period lasted for 15 days. As incubation progressed, incubation‐bout duration had no significant difference through the early, middle and late stages. However, during the 15‐day nestling period, feeding frequency of parents showed a significant difference among stages, with the highest feeding frequency at the late stage. The length of brooding bouts decreased as the nestlings' age increased. Our observations provide detailed records on the nest, nestlings, and breeding behavior of this endangered laughingthrush for the first time. Based on the known information, we emphasized the importance of alpine bamboo forests for the conservation of this laughingthrush. Moreover, we recommend minimizing anthropogenic disturbance in its breeding ground, and prioritizing a comprehensive survey to assess the current population status in its distribution.

## Graphical abstract

Breeding biology of the poorly known Biet's Laughingthrush (*Ianthocincla bieti*) was studied for the first time ever. Two active nests were located, described and monitored. The breeding traits of Biet's Laughingthrush meet the life‐history theory predictions, exhibiting high nest attentiveness, incubating and nestling period extended, and high nesting success.

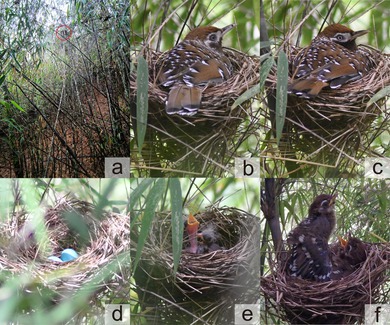

## 1 Introduction

Studies on breeding biology and behavior are fundamental to understand life history evolution and the conservation of rare species (Stutchbury and Morton 2008; Martin 2015; Nagy et al. 2019; Wang et al. 2023). Under the current scenario of global climate change, life history parameters are crucial variables in predictive modeling of species fate (Urban et al. 2016). However, information on the life history and in particular breeding biology of laughingthrushes remains scarce or entirely lacking for most species (del Hoyo et al. 2007). Laughingthrushes are classified within the family Leiothrichidae with 133 species and 16 genera according to the IOC World Bird List (Gill et al. 2023). A representative group is the genus *Ianthocincla*, comprising eight forest‐dwelling understory laughingthrushes based on previous phylogenetic research (Cibois et al. 2018; Cai et al. 2019). They are typically characterized by brownish or grayish‐brown plumage with blackish subterminal bars, and in some species, dot‐like white tips on the upperparts. To date, with the exception of the Snowy‐cheeked Laughingthrush (*I. sukatschewi*) (Wang et al. 2011), almost no scientific accounts have been published on the ecology of the remaining seven species (del Hoyo et al. 2007).

Among these seven species, the Biet's Laughingthrush (*I. bieti*) *was* first described in 1897 from a single unsexed specimen. This elusive bird has drawn scientific attention due to its extremely restricted range and striking plumage, a blend of chestnut, black, and white speckles, unique among all laughingthrushes (Oustalet 1897). It occurs mainly in undisturbed habitats, including mixed forest, thickets, spruce‐fir, and fir‐rhododendron forest, particularly those with dense bamboo understory, within the upper temperate and subalpine zones from 2500 to 4270 m, and possibly to 4570 m in southwestern China (BirdLife International 2001).

The population size and distribution of the Biet's Laughingthrush remain poorly documented. Historically, twelve localities were known for this species, but since 1980 only two were confirmed to hold individuals (BirdLife International 2001). Due to the species' elusive nature, a systematic survey in Yunnan was conducted once, between 2011 and 2012, by zoologist Han Lianxian from Southwest Forestry University (pers. comm.). That survey reconfirmed Ludian and Laojun Mountain as occupied sites, which held the largest populations among the eight surveyed, both located in relatively inaccessible areas. In addition, three new localities were discovered around The Old Town of Lijiang, while two other surveyed sites in the same vicinity no longer supported the species, likely due to heavy trapping pressure. In Laojun Mountain with less trapping pressure, the population is assumed stable but lacks long‐term monitoring. In Sichuan, the species is observed at three localities, with approximately six individuals recorded at each site (Mr. Fu Man, pers. comm., 2024). However, most of these localities were discovered occasionally, and among the total nine existing localities, only five of them are located in the national park or nature reserve.

Recently, a study used niche modeling to predict its potential distribution, indicating that suitable habitat is confined to the southeastern Qinghai–Tibet Plateau (Li et al. 2023). However, there is still a major knowledge gap regarding fine‐scale habitat characteristics and breeding details, which are crucial for its conservation, as the species is classified as Vulnerable on the IUCN Red List (IUCN 2017). As an endemic species to China, it is also listed in category I of the National Key Protected Wild Bird (https://www.forestry.gov.cn/c/www/lczc/90131.jhtml, accessed on 18 September 2024).

The objective of this study was to provide fundamental life‐history information on this poorly known species, including breeding parameters, nest‐site characteristics and breeding‐related behaviors, aiming to raise conservation concerns for this laughingthrush. An additional objective was to compare its breeding ecology with that of other Leiothrichidae species that nest in bamboo habitats within China.

## 2 Methods

### 2.1 Study Area

The Laojun Mountain (26°2′48′′–27°36′36′′ N, 99°1′2′′–99°54′36′′ E) is located in the core area of the Three Parallel Rivers UNESCO World Natural Heritage Site. This region encompasses a vast area with a series of large mountains running north to south along the eastern edge of the Qinghai‐Tibet Plateau (Nie et al. 2024). The Laojun mountain lies between the Jinsha River (upper Yangtze River) and Lancang River (upper Mekong River) (Zhang et al. 2010). The mountain covers about 108,500 hm^2^, with elevations ranging from 1800 to 4513 m (Zhao et al. 2006).

The climate is influenced by the temperate monsoon. The annual temperature averages 12.8°C and the annual average precipitation is 964.7 mm recorded between 1951 and 2017 (Cao et al. 2020). Forest cover exceeds 91.7% (Yu et al. 2012), with an impressive vertical replacement of vegetation from evergreen broad‐leaved and deciduous forests, to coniferous forests, rhododendron shrubs, and alpine meadows towards the summit (Li et al. 2018). Our study was conducted at elevations of 2905–3383 m in mixed coniferous and broad‐leaved forests with bamboo stands understory.

The Spotted Nutcracker ( *Nucifraga caryocatactes* ), Pallas's Squirrel ( *Callosciurus erythraeus* ), Swinhoe's Striped Squirrel ( *Tamiops swinhoei* ), and the Asian Red‐cheeked Squirrel ( *Dremomys rufigenis* ) are main local nest predators.

### 2.2 Fieldwork Procedure

We located a nest of the Biet's Laughingthrush in May 2021, during our survey on avian breeding biology at Laojun Mountain conducted from May to June that year. We returned to the same area in subsequent years to search for additional nests. In April 2024, we found a second nest by following adults carrying nest material. Nest elevations were recorded with a GPS tracking application (2bulu, https://www.2bulu.com/). In 2024, an infrared camera (forsafe H805, Kunshan Jinkehua Electronics Co. Ltd., China) was positioned 1–1.5 m from the nest to document egg‐laying dates and identify potential nest predators. Camera settings were configured to photo‐video mode, taking two photos and recording video for 20 s. We then checked the nest daily to monitor its breeding status, that is, incubating, brooding, or fledging stage. The incubation period was measured as the number of days between the laying of the last egg and the hatching of the last egg (Martin 2002). The nestling period was measured as the period from the hatching of the first nestling until the fledging of at least one bird (Marini et al. 2014).

In order to minimize our disturbance, we placed a GoPro camera (HERO5, GoPro, San Mateo, CA, USA) 1.3 m away from the nest to record the parental behavior. Videos were recorded at 30 frames per second, with each session lasting about 2 h, limited by battery life. As a result, we conducted 1–3 sessions per day, scheduled between 07:00 and 11:00 am and 12:00 and 6:00 pm, depending on weather conditions. The camera was not installed or checked when a parent bird was inside the nest. We used binoculars to make direct observations from a concealed position more than 5 m away. Both observers and equipment were camouflaged to reduce disturbance.

After the nestlings fledged, we measured outer diameter (from edge to edge), inner diameter (cup), outer height (exterior bottom‐to‐top) and inner height (bottom‐to‐top of cup) of the two nests using a sliding caliper. We used an electronic scale (Wuxin Weighing Apparatus Co. Ltd., 20 ± 0.001 g) to weigh the dried nest. In addition, we measured nest height above ground and recorded its distance from the nearest road and water source using a tape measure.

### 2.3 Comparative Study: Bibliographic Review and Synthesis

We collected information on the breeding biology of Leiothrichidae species native to China from the Handbook of the Birds of the World and online databases: Web of Science, Google Scholar and China National Knowledge Infrastructure (CNKI). We performed searches using the common or scientific names of species, in combination with ‘breeding’, ‘reproduction’ or ‘reproductive’ as keywords. For each species, we read the ‘Breeding’ section in the species account of the Handbook or publications found from online resources to determine whether it is a bamboo‐breeder. If it is true, we documented the information of its breeding elevation, nest height, nest structure, clutch size, egg fresh mass, egg size, egg color, egg pattern, incubation period, nestling period in natural habitats, excluding the information obtained under captive conditions. All data were synthesized into a master database. Table 1 presents a comparative summary of these breeding parameters for all Chinese bamboo‐nesting Leiothrichidae species, enabling direct interspecific comparison.

**TABLE 1:** Comparison of breeding characteristics among 22 bamboo‐nesting Leiothrichidae species.

Species	Nest site	Nest			Eggs				Incubation period (d)	Nestling period (d)	References
	Elevation (m a.s.l.)	Height (m)	Structure	Clutch size	Fresh mass (g)	Size (mm)	Color	Speckles			
Elliot's Laughingthrush ( *Trochalopteron elliotii* )	1500–2100	1.5–2 (*n* = 3)	Cup	3.4 (*n* = 18)	5.6 (*n* = 59)	27.1 (*n* = 59) × 19.5 (*n* = 58)	Greenish‐blue	Yes	14.2 (*n* = 13)	15	Opaev et al. (2017); Liu et al. (2021)
Red‐tailed Laughingthrush ( *Trochalopteron milnei* )	1500	2.2 (*n* = 25)	Cup	2.8 (*n* = 12)	6.1 (*n* = 7)	30 × 21 (*n* = 7)	White	Yes	—	15	(Jiang et al. 2007; Yang et al. 2022)
Chestnut‐crowned Laughingthrush ( *Trochalopteron erythrocephalum* )	2000–2600	0.5–2	Cup	2–3 (*n* = 10)	—	—	Blue	Yes	—	—	Acharya and Vijayan (2009)
Red‐winged Laughingthrush ( *Trochalopteron formosum* )	1507–1944	2.8 (1.7–7.5, *n* = 29)	Cup	2.3 (2–3, *n* = 24)	—	29.7 × 21.3 (*n* = 6)	Blue‐green or blue	Yes	14–15 (*n* = 53)	14 (*n* = 23)	Wu et al. (2018)
Silver‐eared Laughingthrush ( *Trochalopteron melanostigma* )	—	—	Cup	2	—	—	Blue	Yes	—	—	del Hoyo et al. (2007)
Rusty‐fronted Barwing ( *Actinodura egertoni* )	—	—	Cup	—	—	—	Blue	Yes	—	—	del Hoyo et al. (2007)
Red‐billed Leiothrix ( *Leiothrix lutea* )	1520	1.4 (*n* = 25)	Cup	3.1 (3–5, *n* = 25)	2.6 (2.4–3, *n* = 3)	20.6 × 15.9 (*n* = 7)	White or blue	Yes	12 (*n* = 1)	10–12	del Hoyo et al. (2007); Zhou et al. (2012)
Emei Shan Liocichla ( *Liocichla omeiensis* )	1450–2150	1.4 (0.6–1.8, *n* = 34)	Cup	2.9 (2–4, *n* = 10)	4.0 (*n* = 7)	24.9 × 17.4 (*n* = 11)	Blue	Yes	~14 (*n* = 29)	~13–14 (*n* = 24)	Fu et al. (2011)
Scarlet‐faced Liocichla ( *Liocichla ripponi* )	—	0.6–1.5	Cup	3	—	—	Blue	Yes	—	—	del Hoyo et al. (2007)
Spot‐breasted Laughingthrush ( *Garrulax merulinus* )	—	—	Cup	2–3	—	—	Blue	—	—	—	del Hoyo et al. (2007)
Lesser Necklaced Laughingthrush ( *Garrulax monileger* )	—	—	Cup	3–5	—	—	Blue	No	—	—	del Hoyo et al. (2007)
Mustached Laughingthrush (*Ianthocincla cineracea*)	—	1–2	Cup	2–5	—	—	Blue	—	12–13	12	del Hoyo et al. (2007)
Giant Laughingthrush (*Ianthocincla maxima*)	2850–2950	4 (2.4–7, *n* = 7)	Bowl	2.2 (2–3, *n* = 6)	7.8–10.0 (*n* = 10)	33.5 × 22.9 (*n* = 10)	Blue	No	—	17–18 (*n* = 2)	Wang et al. (2010)
White‐speckled Laughingthrush (*Ianthocincla bieti*)	2905–3380	3.8, 4.5	Cup or bowl	2	—	—	Blue	No	15 (*n* = 1)	15 (*n* = 1)	This study
Black‐throated Laughingthrush (*Pterorhinus chinensis*)	—	2.1	Cup	3–5	—	—	Blue or white	—	—	—	del Hoyo et al. (2007)
White‐browed Laughingthrush (*Pterorhinus sannio*)	280	5.2 (*n* = 15)	Bowl	3–4 (*n* = 4)	5.1 (*n* = 13)	24.8 × 18.9 (*n* = 13)	Blue or white	—	11–12	12 (*n* = 1)	Zhu et al. (2010)
Masked Laughingthrush (*Pterorhinus perspicillatus*)	30–1250	3.7 (1.7–7.1, *n* = 63)	Bowl	3.8 (3–7, *n* = 47)	6.9 (*n* = 39)	29.1 × 21.3 (*n* = 117)	Blue	No	13–15 (*n* = 10)	12–15 (*n* = 14)	Guo et al. (2022)
Greater Necklaced Laughingthrush (*Pterorhinus pectoralis*)	1438	6	Cup	3–7	—	—	Blue	—	—	—	del Hoyo et al. (2007); Lu et al. (2024)
White‐throated Laughingthrush (*Pterorhinus albogularis*)	1546–1987	2.3 (*n* = 4)	Cup	3 (*n* = 3)	—	—	Blue	Yes	—	—	Fan et al. (2024)
Gray‐sided Laughingthrush (*Pterorhinus caerulatus*)	—	1–3.7	Cup	2–3	—	—	Blue	—	—	—	del Hoyo et al. (2007)
Buffy Laughingthrush (*Pterorhinus berthemyi*)	1500	2.4 (*n* = 12)	Cup	3.8 (*n* = 9)	—	—	Blue	No	—	—	Yang et al. (2022)
Rusty Laughingthrush (*Pterorhinus poecilorhynchus*)	—	—	Cup	2–3	—	—	Blue	No	—	—	del Hoyo et al. (2007)

*Note:* — indicates no data exists.

### 2.4 Data Analysis

We used the Shapiro–Wilk test to check the normality of incubation bouts, egg‐turning rate, brooding bouts and feeding frequency. For non‐paired data, if the sample size was small or the data were not normally distributed, we employed the non‐parametric Wilcoxon rank sum test to compare males and females. If the paired data were normally distributed, paired‐sample t‐tests were employed; otherwise, Wilcoxon signed rank tests were employed (Liang et al. 2017). We subdivided the incubation period into three stages (Goulding and Martin 2010; Lin et al. 2010): early (Days 1–5), middle (Days 6–10) and late (Days 11–15). The nestling period was also subdivided into three stages (Lin et al. 2010): early (Days 1–5 post‐hatching), middle (Days 6–10), and late (Days 11–15). Incubation bouts, turning eggs, brooding bouts, and feeding frequency were compared between sexes and across stages. Analysis of variance (ANOVA) was used to test for differences in incubation bouts and feeding frequency among different stages when data were normally distributed. Kruskal–Wallis rank sum tests were used for two‐by‐two comparisons when the data were not normally distributed. R was used for statistical tests (R Core Team 2023). All statistical tests were two‐tailed. Results are presented as mean ± SD.

## 3 Results

### 3.1 Nest Site and Nest

**FIGURE 1:**
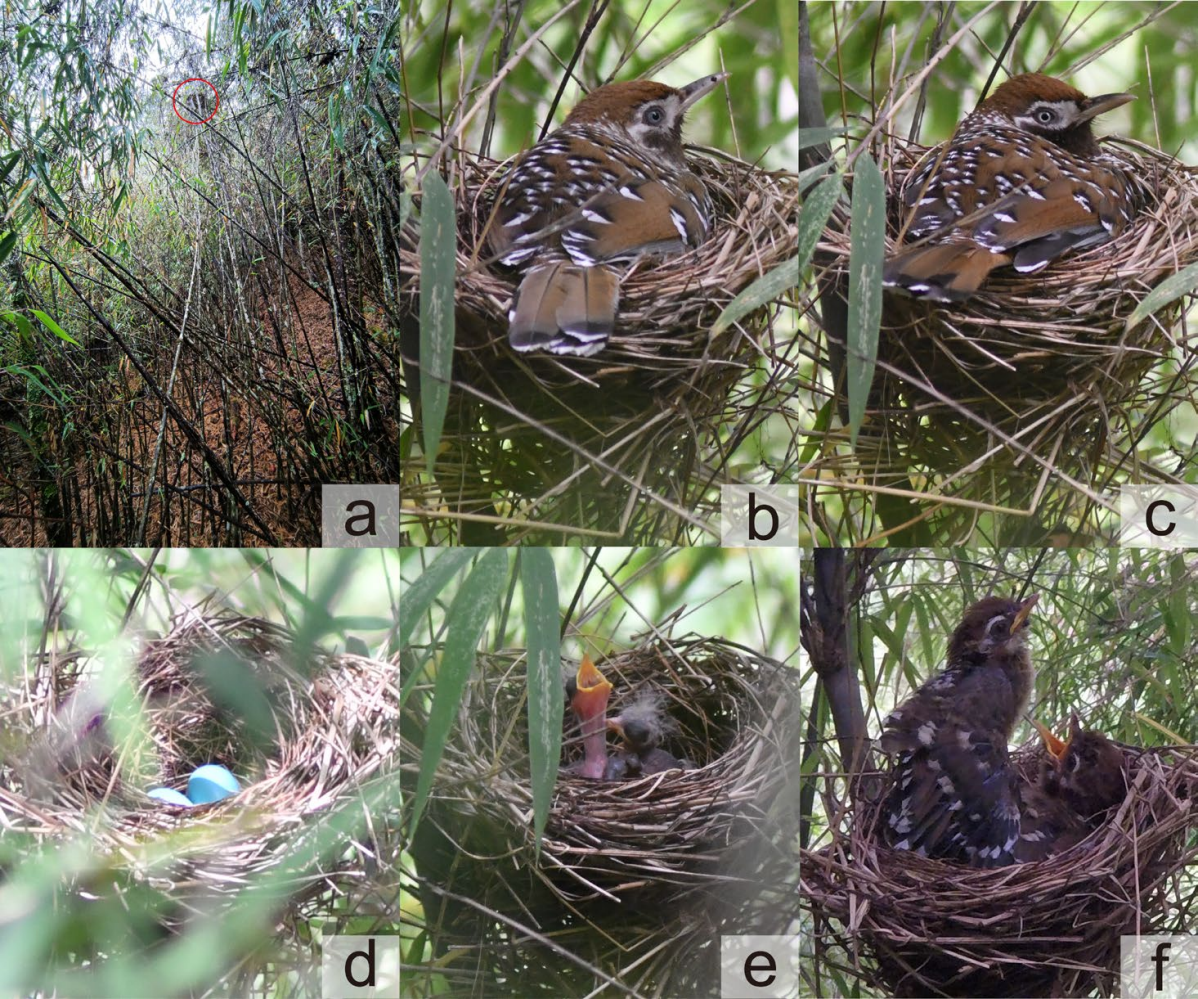
Images showing the nesting traits of the Biet's Laughingthrush at Laojun Mountain, China. (a) Nest location (circled) in dense bamboo forest at 3380 m elevation, (b) brooding adult male (iris pale blue), (c) brooding adult female (iris white), (d) eggs, (e) 3‐day‐old nestlings observed in May, and (f) 12‐day‐old nestlings observed in June.

Nests were generally found in April and May. The nesting season extends from April to June. In May 2021, we found a nest of the Biet's Laughingthrush containing two nestlings, which fledged after 3 days. The nest was located 4.5 m above the ground in a bamboo thicket at 3380 m elevation; it was constructed using branches from several standing bamboos, which gave good support for the nest (Figure 1a). In April 2024, we found another active nest under construction, which was completed within 5 days. The interval between nest completion and initial egg laying was about 4 days. Unlike the 2021 nest, this one was built into the clustered nodes of a single *Fargesia* bamboo, 3.7 m above the ground at 2905 m elevation. Intensive bamboo harvesting for agricultural use and livestock grazing was recorded within the species' habitat during the study period.

Both nests were bowl‐or cup‐shaped with a vertical upward opening. The inner lining of the nests was composed of dry grass stems, and the exterior surface contained fine bamboo branches, bamboo leaves and moss. They were 11.1 and 9.4 cm in inner diameter, 16.2 and 12.2 cm in outer diameter, 2.4 and 5.2 cm in inner height, 6.9 and 9.5 cm in outer height, respectively. The dry nest mass was 49.6 g and 33.6 g. Nests are 14.8 m and 13.5 m away from the footpath and 32.6 m and 6 m away from the water source respectively. The cover near the nest was dense. In addition to *Fargesia* bamboo, the surrounding vegetation included trees and shrubs such as *Cunninghamia lanceolata* and *Sambucus javanica*. The parents foraged primarily on the ground covered with fine fallen leaves.

### 3.2 Eggs and Incubation

In 2024, the clutch consisted of two oval, pale blue eggs without speckles (Figure 1d). We determined that the adult with a white iris was the female (egg‐laying), while the individual with a blue iris was the male. The female laid one egg per day between 07: 30 and 09: 30 am. The incubation period lasted 15 days (*n* = 1). During incubation, both parents took turns sitting on the nest for most of the day (Figure 1b,c). Nest relief was sometimes preceded by a short vocal exchange between the pair. When the female approached the nest, she gave a call, prompting the incubating male to respond and leave the nest immediately. The same pattern occurred when the male returned to relieve the female. Eggs were exposed only briefly during changeovers, with intervals averaging 0.6 min (SD = 1.2, range 0.1–6.2, *n* = 44). During incubation, parents turned the eggs on average once every 19.43 min (SD = 12.06, range 8.6–69.3, *n* = 305), that is, every 33.49 min by the female, while every 77.65 min by the male. Egg‐turning was significantly more frequent in females than in males (*W* = 132, *p* = 0.0001). Male and female had no difference in length of incubation bouts (*t* = −0.834, df = 88.2, *p* = 0.41). In addition, incubation bouts in early (*t* = 0.467, df = 37.9, *p* = 0.643), middle (*t* = −0.381, df = 25.3, *p* = 0.706), and late stages (*t* = −0.618, df = 20, *p* = 0.544) showed no significant differences between sexes. Across stages, overall incubation bout length did not differ significantly (*F* = 1.553, df = 2, *p* = 0.217). The sexes show differences in territorial behavior, with the male calling more frequently than the female during incubation. Both sexes emit alarm calls and chase conspecifics near the nest site. Furthermore, we observed once the male drove away the Pallas's squirrel ( *C. erythraeus* ) that were active diagonally above the nest—chasing them from small branches to the arbor, circling around the trunk until the squirrels were driven out of the area, approximately 10 m away from the nest.

### 3.3 Parental Care and Growth of Nestlings

**FIGURE 2:**
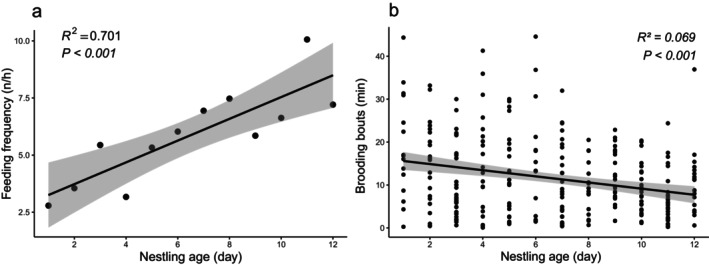
(a) Fluctuation in feeding frequency of nestlings. (b) Fluctuation in brooding bouts of the Biet's Laughingthrush.

The nestling period is 15 d (*n* = 1). The two eggs hatched on 20 May, and both nestlings fledged on 3 June in 2024. Both adults provisioned, brooded, and cleaned the nest throughout the nestling period. During this phase, 2627 min of video and 1977 min of observation were made. We recorded 464 provisioning events, and the inter‐provisioning interval averaged 9.5 min (SD 8.7, range 0.1–46.0, *n* = 438). The female fed 2.97 ± 1.12 times per hour, the male fed 2.86 ± 1.02 times per hour. Feeding frequency increased with nestling age (Figure 2a). No sexual differences were found in overall provisioning frequency (*W* = 76, *p* = 0.84), or during the early (*t* = −0.06, df = 4, *p* = 0.96), middle (*t* = −0.07, df = 4, *p* = 0.95), or late stages (*W* = 1, *p* = 1). However, the provisioning frequency differed significantly among stages (Kruskal–Wallis chi‐squared = 15.287, df = 2, *p* = 0.0005), with the late stage being the highest. Food items included various invertebrates such as earthworms, caterpillars, larvae etc. We observed once the parent tore a large caterpillar into pieces by shaking and hitting it against the ground. Provisioning was always followed by brooding, even when the nestlings were feathered or close to fledging (Figure 1e,f). On average, each brooding bout was 9.3 min (SD = 9.3, range 0.1–44.6, *n* = 274) with no sexual difference (*W* = 9148.5, *p* = 0.789). Brooding bout duration decreased as nestlings aged (Figure 2b). Parent birds swallowed (*n* = 120) or sometimes carried away (*n* = 2) fecal sacs.

We recorded two cases of anti‐predator behavior, preventing squirrel predation on nestlings. On one occasion, the female returned with food in her mouth and found a squirrel in the understory. While the female attempted to drive it away, the brooding male joined in and chased the squirrel. Frightened, the squirrel ran immediately, leaving a 10 m radius around the nest. Then the female returned to the nest to feed the nestlings. On another occasion, one adult bird quickly chased the squirrel along a thick fallen timber. At hatching, nestlings were pink with a few gray downy feathers on the head, back, and wings. They started extending their necks to beg for food by the second day (Figure 1e). By the fourth day, the primary and secondary wing feathers budded up the skin. On the sixth day, the eyes had started to open, and the feathers had grown swiftly. Light yellow feathers cover the midline of the neck, both sides of the chest, and the abdomen by the eighth day. By Day 11, plumage resembled that of adults, except for incomplete feathers of the periocular region and ungrown tail feathers. By Day 12, nestlings were able to preen and flap their wings (Figure 1f).

### 3.4 Breeding Comparisons of 22 Bamboo‐Nesting Leiothrichidae

Although there are few accounts existing in the literature, we summarize the breeding information of Leiothrichidae species, focusing on the bamboo breeders which include 22 species (Table 1).

Most species nested at mid‐ to high elevations between 1500 and 3000 m a.s.l., with nest heights typically ranging from less than 1 m to over 6 m above ground. The majority of species laid 2–4 eggs per clutch, while a few (e.g., *Pterorhinus pectoralis*) produced as many as seven. Eggs were generally blue to bluish or greenish‐blue, often with speckles, though four species could lay white eggs and six species laid spotless eggs. Egg size ranged approximately from 20 to 33 mm in length and 15 to 23 mm in width, and fresh mass ranged between 2 and 6 g when reported. Incubation periods were typically 12–15 days, followed by nestling periods lasting 10–15 days. High‐elevation taxa, including *Ianthocincla maxima* and *I. bieti*, tend to have smaller clutch sizes, larger eggs and longer nestling periods compared with low‐ and mid‐elevation congeners.

## 4 Discussion

### 4.1 Nest Site and Nest

The nests of the Biet's Laughingthrush were built 3.7–4.5 m above the ground within dense bamboo stands at elevations between 2905 and 3380 m. Dense bamboo vegetation offers effective concealment, and the elevated nest placement likely helps reduce predation risk—a major cause of nest failure in most laughingthrush (Wu et al. 2018; Fu et al. 2011; Yang et al. 2022). Similar nest placement has been reported in other bamboo‐nesting laughingthrushes, such as *I. maxima* , indicating a convergent strategy for camouflage within alpine bamboo habitats. The timing of nesting (April–May) coincides with the early rainy season in the study area, when insect availability increases, providing abundant food for adults and nestlings (Grames et al. 2023). However, anthropogenic activities, particularly at higher elevations, may negatively affect the breeding success of these birds (Wang et al. 2019).

### 4.2 Incubation

Our observations indicated that Biet's Laughingthrush were socially monogamous. The incubation behavior of the Biet's Laughingthrush shows a high level of parental coordination and investment. Both sexes alternated incubation duties with similar bout durations, but females turned the eggs significantly more frequently than males. Periodically egg turning enhances embryo development by regulating temperature, facilitating respiratory gas exchange (Boulton and Cassey 2012; Portugal et al. 2014; Pešková et al. 2025), and the higher rate by females suggests a greater direct role in egg maintenance. Both sexes were involved in nest defense and territory maintenance, consistent with patterns observed in *Trochalopteron elliotii* and *Garrulax sukatschewi* (Wang et al. 2011; Opaev et al. 2017). The active defense against a Pallas's squirrel ( *C. erythraeus* ) demonstrates that predation risk remains a significant selective pressure shaping vigilant parental behavior.

### 4.3 Parental Care

Both adults contributed equally to feeding, brooding, and nest sanitation throughout the 15‐day nestling period, with no sexual difference in provisioning frequency. Such balanced parental roles are typical of passerines inhabiting harsh montane environments, where biparental cooperation maximizes offspring survival (Cockburn 2006; Long et al. 2022). The increase in feeding rate with nestling age reflects the rising energetic demands of growing chicks (Cauchard et al. 2021). The observed prey handling—tearing large caterpillars before feeding—suggests behavioral flexibility to accommodate prey size and nestlings' developmental stage, a pattern also reported in *Leiothrix lutea* (Zhou et al. 2012). The nestling period (15 days) exceeded that of most mid‐ and low‐elevation laughingthrushes, yet remained shorter than that reported for *Ianthocincla maxima*.

### 4.4 Comparative Summary

Our comparative summary of 22 bamboo‐nesting Leiothrichidae species reveals ecological patterns in breeding traits along elevational gradients. These species can be broadly divided into high‐elevation breeders (mostly above 1500 m a.s.l.) and low‐elevation breeders. Species in the first group, such as the Giant Laughingthrush ( *I. maxima* ), Elliot's Laughingthrush ( *Trochalopteron elliotii* ), Red‐winged Laughingthrush ( *T. formosum* ), and Chestnut‐crowned Laughingthrush ( *T. erythrocephalum* ), typically lay small clutches of two to three eggs, have prolonged incubation and nestling periods, and exhibit high parental investment patterns consistent with life‐history theory predictions for alpine breeding birds (Badyaev and Ghalambor 2001). The Biet's Laughingthrush provides a representative example: it lays a clutch of only two eggs, both the incubation and nestling periods last 15 days which is 2–3 days longer than the low‐elevation breeders (Table 1). At least one adult remains on or near the nest throughout the breeding stages, and both sexes participate in strong nest defense. In contrast, low‐elevation species, such as the Masked Laughingthrush (*Pterorhinus perspicillatus*) and Greater Necklaced Laughingthrush ( *P. pectoralis* ), produce larger clutches (3–7 eggs), have shorter nestling periods, and show more flexible parental care strategies, reflecting the generally warmer and more stable environments in which they breed.

### 4.5 Conservation Implications

The Biet's Laughingthrush, despite being listed as Vulnerable on the IUCN Red List and classified as a Class I nationally protected species in China, has a population size and current distribution that remain poorly understood. Recent field surveys indicate that the species now persists at only a few isolated localities in northwestern Yunnan and southwestern Sichuan, and several historical sites may already have experienced local extirpation. Given these records are from different mountains, far from each other, thus we propose to carry out a comprehensive survey of its distribution and population status for this species.

Previous studies demonstrate that bamboo stands in southwest China have been under sustained anthropogenic pressure for decades (Campbell 1984). Although the implementation of the Natural Forest Protection Program (NFPP) in the late 1990s has effectively reduced large‐scale logging, small‐scale bamboo harvesting and grazing still occur locally, continuing to threaten the species that is dependent on dense bamboo understory within montane broadleaf and coniferous forests (Chaves et al. 2017; Tian et al. 2025). To ensure the long‐term survival of *I. bieti*, we recommend strengthening protection and habitat management in key sites such as Laojun Mountain and Ludian, where the species has been recently confirmed. Access to nesting habitats during the breeding season should be restricted in core conservation zones to minimize disturbance. In addition, studies integrating habitat modeling, demographic monitoring, and local community engagement are essential to identify and manage critical habitats effectively.

## 5 Conclusions

We provide the first account of the breeding biology of the Biet's Laughingthrush, filling important gaps in our knowledge of this vulnerable species. The life‐history traits of Biet’s Laughingthrush represent the typical of high‐elevation breeders, including small clutch size, extended incubation and nestling periods, and high parental investment. These traits reflect adaptations to the cold and unpredictable conditions of these habitats. The study also highlights the significant role of bamboo forests in supporting this species, offering essential food resources and protection from predators. Despite its vulnerable status, the species' population size and distribution remain poorly understood and require urgent investigation. To ensure the conservation of this species, we recommend a comprehensive survey of its population status and the prohibition of bamboo harvesting practices during the breeding season in the Laojun Mountain.

## Author Contributions

**Caiping Chen:** data curation (lead), formal analysis (lead), writing – original draft (lead), writing – review and editing (lead). **Shixiang Fan:** data curation (equal), investigation (lead), methodology (lead), writing – review and editing (supporting). **Gang Song:** writing – review and editing (supporting). **Yanhua Qu:** funding acquisition (lead), writing – review and editing (supporting). **Xu Luo:** conceptualization (lead), project administration (lead), resources (lead), supervision (lead), writing – review and editing (equal).

## Funding

This work was supported by The National Natural Science Foundation of China (U23A20162) and the Talent Nurturing Program of Yunnan Province.

## Ethics Statement

The study was carried out under the Institutional Animal Care and Use Committee at Southwest Forestry University. No nests or nestlings were harmed during our fieldwork.

## Conflicts of Interest

The authors declare no conflicts of interest.

## Supporting information


**Data S1:** ece372628‐sup‐0001‐DataS1.csv.

**Data S2:** ece372628‐sup‐0002‐DataS2.r.

**Data S3:** ece372628‐sup‐0003‐DataS3.r.

**Data S4:** ece372628‐sup‐0004‐DataS4.csv.

## Data Availability

Necessary data for this study is all included in the main manuscript. All the required data are uploaded as Data [Link], [Link], [Link], [Link].

## References

[ece372628-bib-0001] Acharya, B. K. , and L. Vijayan . 2009. “Breeding Bird Community and Their Nesting Characteristics in the Teesta Valley of Sikkim.” Journal of Hill Research 22, no. 1: 1–8.

[ece372628-bib-0002] Badyaev, A. V. , and C. K. Ghalambor . 2001. “Evolution of Life Histories Along Elevational Gradients: Trade‐Off Between Parental Care and Fecundity.” Ecology 82, no. 10: 2948–2960.

[ece372628-bib-0003] BirdLife International . 2001. Threatened Birds of Asia: The BirdLife International Red Data Book. BirdLife International.

[ece372628-bib-0004] Boulton, R. L. , and P. Cassey . 2012. “How Avian Incubation Behaviour Influences Egg Surface Temperatures: Relationships With Egg Position, Development and Clutch Size.” Journal of Avian Biology 43, no. 4: 289–296.

[ece372628-bib-0005] Cai, T. , A. Cibois , P. Alström , et al. 2019. “Near‐Complete Phylogeny and Taxonomic Revision of the World's Babblers (Aves: Passeriformes).” Molecular Phylogenetics and Evolution 130: 346–356. 30321696 10.1016/j.ympev.2018.10.010

[ece372628-bib-0006] Campbell, J. J. 1984. “Giant Panda Conservation and Bamboo Forest Destruction.” In Studies in Environmental Science, vol. 25, 599–616. Elsevier.

[ece372628-bib-0007] Cao, R. , D. Yin , K. Tian , et al. 2020. “Response of Radial Growth of *Abies georgei* and *Tsuga dumosa* to Climate Change at Upper Distributional Limits on Laojun Mountain, Lijiang, Yunnan, China.” Acta Ecologica Sinica 40, no. 17: 6067.

[ece372628-bib-0008] Cauchard, L. , E. I. Macqueen , R. Lilley , P. Bize , and B. Doligez . 2021. “Inter‐Individual Variation in Provisioning Rate, Prey Size and Number, and Links to Total Prey Biomass Delivered to Nestlings in the Collared Flycatcher (*Ficedula albicollis*).” Avian Research 12, no. 1: 15.

[ece372628-bib-0009] Chaves, W. A. , K. E. Sieving , and R. J. Fletcher Jr. 2017. “Avian Responses to Reduced‐Impact Logging in the Southwestern Brazilian Amazon.” Forest Ecology and Management 384: 147–156.

[ece372628-bib-0010] Cibois, A. , M. Gelang , P. Alström , et al. 2018. “Comprehensive Phylogeny of the Laughingthrushes and Allies (Aves, Leiothrichidae) and a Proposal for a Revised Taxonomy.” Zoologica Scripta 47, no. 4: 428–440.

[ece372628-bib-0011] Cockburn, A. 2006. “Prevalence of Different Modes of Parental Care in Birds.” Proceedings of the Royal Society B: Biological Sciences 273, no. 1592: 1375–1383. 10.1098/rspb.2005.3458 PMC1560291 16777726

[ece372628-bib-0012] del Hoyo, J. , A. Elliott , and D. Christie . 2007. “Handbook of the Birds of the World: Vol. 12. Picathartes to Tits and Chickadees Lynx Edicions.”

[ece372628-bib-0013] Fan, B. , Y. Zhang , P. Li , J. Chen , Y. Fu , and W. Qiong . 2024. “Breeding Ecology of *Pterorhinus albogularis* .” Sichuan Journal of Zoology 43, no. 5: 505–511. https://link.cnki.net/urlid/51.1193.q.20240620.1724.020.

[ece372628-bib-0014] Fu, Y. , S. D. Dowell , and Z. Zhang . 2011. “Breeding Ecology of the Emei Shan Liocichla (*Liocichla omeiensis*).” Wilson Journal of Ornithology 123, no. 4: 748–754.

[ece372628-bib-0015] Gill, F. , D. Donsker , and P. Rasmussen . 2023. “IOC World Bird List (Version 15.1).” International Ornithologists' Union. https://www.worldbirdnames.org.

[ece372628-bib-0016] Goulding, W. , and T. E. Martin . 2010. “Breeding Biology of the Golden‐Faced Tyrannulet ( *Zimmerius chrysops* ) in Venezuela.” Wilson Journal of Ornithology 122, no. 4: 689–698.

[ece372628-bib-0017] Grames, E. M. , G. A. Montgomery , C. Youngflesh , M. W. Tingley , and C. S. Elphick . 2023. “The Effect of Insect Food Availability on Songbird Reproductive Success and Chick Body Condition: Evidence From a Systematic Review and Meta‐Analysis.” Ecology Letters 26, no. 4: 658–673. 36798988 10.1111/ele.14178

[ece372628-bib-0018] Guo, W. , B. Lin , Z. Hu , S. Tan , J. Tian , and C. Wang . 2022. “Nest Predation, Brood Parasitism and the Reproductive Success of the Masked Laughingthrush *Garrulax perspicillatus* in the Rural Habitat of Central China.” Avian Biology Research 15, no. 2: 61–67.

[ece372628-bib-0019] IUCN . 2017. “Biet's Laughingthrush (*Ianthocincla bieti*) (Amended Version of 2017 Assessment).” The IUCN Red List of Threatened Species. https://www.iucnredlist.org/species/22715675/118588681.

[ece372628-bib-0020] Jiang, Y. , Y. Sun , and W. Liang . 2007. “Notes on the Breeding Nest of the Red‐Tailed Laughingthrush (*Garrulax milnei*) in Kuankuoshui, Guizhou.” Chinese Journal of Zoology 42, no. 2: 16. 10.13859/j.cjz.2007.02.004.

[ece372628-bib-0021] Li, B. , C. Liang , P. Song , et al. 2023. “Threatened Birds Face New Distribution Under Future Climate Change on the Qinghai‐Tibet Plateau (QTP).” Ecological Indicators 150: 110217.

[ece372628-bib-0022] Li, Y. , J. He , C. Li , B. Xu , and X. Xie . 2018. “A Study of the Relationship Between the Pollen in the Surface Sediments and the Vegetation From the Laojun Mountain of Lijiang, Yunnan Province, Southwest China.” Acta Micropalaeontologica Sinica 35: 51–64.

[ece372628-bib-0023] Liang, D. , G. Gao , L. Han , and X. Luo . 2017. “Breeding Biology of Fire‐Tailed Myzornis (*Myzornis pyrrhoura*) in an Alpine Environment in Southwestern China.” Wilson Journal of Ornithology 129, no. 3: 568–575.

[ece372628-bib-0024] Lin, S. , F. Lu , F. Shan , et al. 2010. “Breeding Biology of the Taiwan Barbet (*Megalaima nuchalis*) in Taipei Botanical Garden.” Wilson Journal of Ornithology 122, no. 4: 681–688.

[ece372628-bib-0050] Liu, P. , X. Qin , and F. Shang . 2021. “Breeding Biology of Two Coexisting Laughingthrush Species in Central China.” Pakistan Journal of Zoology 53, no. 6: 2203–2209.

[ece372628-bib-0025] Long, X. , Y. Liu , A. Liker , F. J. Weissing , J. Komdeur , and T. Székely . 2022. “Does Ecology and Life History Predict Parental Cooperation in Birds? A Comparative Analysis.” Behavioral Ecology and Sociobiology 76, no. 7: 92.

[ece372628-bib-0026] Lu, W. , J. Li , Y. Lei , J. Duan , and K. Luo . 2024. “Observation of Interspecific Feeding by White‐Browed Shrike‐Babbler (*Pteruthius aeralatus*) of Brood‐Parasitic Nestlings of Chestnut‐Winged Cuckoo (*Clamator coromandus*) in a Nest of Greater Necklaced Laughingthrush (*Pterorhinus pectoralis*).” Ecology and Evolution 14, no. 5: e11465. 38783848 10.1002/ece3.11465 PMC11112293

[ece372628-bib-0027] Marini, M. A. , M. M. Vasconcelos , and Y. Lobo . 2014. “Reproductive Biology and Territoriality of the Wedge‐Tailed Grass‐Finch ( *Emberizoides herbicola* ) (Aves: Passeriformes).” Bioscience Journal 30: 853–862.

[ece372628-bib-0028] Martin, T. E. 2002. “A New View of Avian Life‐History Evolution Tested on an Incubation Paradox.” Proceedings of the Royal Society of London. Series B: Biological Sciences 269, no. 1488: 309–316. 10.1098/rspb.2001.1879 PMC1690888 11839200

[ece372628-bib-0029] Martin, T. E. 2015. “Age‐Related Mortality Explains Life History Strategies of Tropical and Temperate Songbirds.” Science 349, no. 6251: 966–970. 26315435 10.1126/science.aad1173

[ece372628-bib-0030] Nagy, J. , M. E. Hauber , I. R. Hartley , and M. C. Mainwaring . 2019. “Correlated Evolution of Nest and Egg Characteristics in Birds.” Animal Behaviour 158: 211–225.

[ece372628-bib-0031] Nie, J. , S. Fan , and X. Luo . 2024. “First Account of the Breeding Biology of Indian Blue Robin (*Larvivora brunnea*) in Southwest China.” Animals 14: 39. 10.3390/ani14010039 PMC10778059 38200770

[ece372628-bib-0032] Opaev, A. , M. Liu , and Z. Kang . 2017. “Behavioral Ecology of Elliot's Laughingthrush (*Trochalopteron* (*Garrulax*) *Elliotii*; Timaliidae): I. Breeding Biology and Social Behavior.” Biology Bulletin 44: 1090–1099.

[ece372628-bib-0033] Oustalet, É. 1897. “Description de Deux Espèces Nouvelles d'Oiseaux du Yun‐Nan.” Bulletin du Museum Paris 3: 162–163.

[ece372628-bib-0034] Pešková, L. , M. Sládeček , M. Šálek , et al. 2025. “Egg Turning Rates in Birds: A Review of Recording Methods and the Influence of Egg Composition and Developmental Maturity.” Ornithology: ukaf045. 10.1093/ornithology/ukaf045.

[ece372628-bib-0035] Portugal, S. J. , G. Maurer , G. H. Thomas , M. E. Hauber , T. Grim , and P. Cassey . 2014. “Nesting Behaviour Influences Species‐Specific Gas Exchange Across Avian Eggshells.” Journal of Experimental Biology 217, no. 18: 3326–3332. 25232199 10.1242/jeb.103291 PMC4179895

[ece372628-bib-0036] Stutchbury, B. J. , and E. S. Morton . 2008. “Recent Advances in the Behavioral Ecology of Tropical Birds.” Wilson Journal of Ornithology 120, no. 1: 26–37.

[ece372628-bib-0037] Tian, H. , Y. Zeng , Z. Zhang , M. Lu , and W. Wei . 2025. “Grazing‐Induced Habitat Degradation: Challenges to Giant Panda Survival Resulting From Declining Bamboo and Soil Quality.” Animals 15, no. 2: 202. 39858202 10.3390/ani15020202 PMC11758315

[ece372628-bib-0038] Urban, M. C. , G. Bocedi , A. P. Hendry , et al. 2016. “Improving the Forecast for Biodiversity Under Climate Change.” Science 353, no. 6304: aad8466. 27609898 10.1126/science.aad8466

[ece372628-bib-0039] Wang, D. , W. Zhang , S. Yang , and X. Y. L. Richter . 2023. “Sex Differences in Avian Parental Care Patterns Vary Across the Breeding Cycle.” Nature Communications 14, no. 1: 6980. 10.1038/s41467-023-42767-5 PMC10620184 37914691

[ece372628-bib-0040] Wang, J. , C. Jia , S. Tang , Y. Fang , and Y. Sun . 2010. “Breeding of the Giant Laughingthrush (*Garrulax maximus*) at Lianhuashan, Southern Gansu, China.” Wilson Journal of Ornithology 122, no. 2: 388–391.

[ece372628-bib-0041] Wang, J. , C. Jia , S. Tang , Y. Fang , and Y. Sun . 2011. “Breeding Biology of the Snowy‐Cheeked Laughingthrush (*Garrulax sukatschewi*).” Wilson Journal of Ornithology 123: 146–150.

[ece372628-bib-0042] Wang, X. , J. Huang , T. A. Connor , et al. 2019. “Impact of Livestock Grazing on Biodiversity and Giant Panda Habitat.” Journal of Wildlife Management 83, no. 7: 1592–1597.

[ece372628-bib-0043] Wu, Y. , C. Kong , M. Xiang , and Y. Fu . 2018. “Breeding Ecology of *Trochalopteron formosum* in Sichuan Laojunshan National Nature Reserve.” Sichuan Journal of Zoology 37: 578–584.

[ece372628-bib-0044] Yang, C. , X. Yao , Y. Cai , G. Li , and W. Liang . 2022. “Breeding Ecology of Two Sympatric Laughingthrushes (*Trochalopteron milnei* and *Garrulax berthemyi* ) in Southwestern China.” Avian Research 13: 100024.

[ece372628-bib-0045] Yu, Y. , X. Wu , and Z. Yang . 2012. Comprehensive Ecosystem Survey and Management Demonstration Study of Lijiang Laojun Mountain. Yunnan Science and Technology Press.

[ece372628-bib-0046] Zhang, Y. , D. Zhou , Q. Zhao , T. Zhou , and K. D. Hyde . 2010. “Diversity and Ecological Distribution of Macrofungi in the Laojun Mountain Region, Southwestern China.” Biodiversity and Conservation 19: 3545–3563.

[ece372628-bib-0047] Zhao, Q. , Y. Zhang , L. Yuan , et al. 2006. “Study on the Resources and Utilization of Medical Fungi in Laojun Mountain.” Journal of Microbiology 26, no. 4: 85–88.

[ece372628-bib-0048] Zhou, M. , S. Yuan , C. Zhou , et al. 2012. “The Preliminary Study on Breeding Habit of *Leiothrix lutea* at Laojunshan Nature Reserve, Sichuan.” Sichuan Journal of Zoology 31, no. 6: 965–969.

[ece372628-bib-0049] Zhu, F. , C. Zhou , Z. Yang , and X. Li . 2010. “Observation on the Breeding Habit of *Garrulax sannio* in Nanchong, Sichuan.” Chinese Journal of Zoology 45, no. 4: 150–155. 10.13859/j.cjz.2010.04.009.

